# Chronic enrichment affects nitrogen removal in tidal freshwater river and estuarine creek sediments

**DOI:** 10.1002/jeq2.20674

**Published:** 2025-01-13

**Authors:** Anne Margaret H. Smiley, Suzanne P. Thompson, Michael F. Piehler

**Affiliations:** ^1^ Institute for the Environment, University of North Carolina at Chapel Hill Chapel Hill North Carolina USA; ^2^ Department of Earth Marine and Environmental Sciences University of North Carolina at Chapel Hill Chapel Hill North Carolina USA

## Abstract

Population growth in coastal areas increases nitrogen inputs to receiving waterways and degrades water quality. Wetland habitats, including floodplain forests and marshes, can be effective nitrogen sinks; however, little is known about the effects of chronic point source nutrient enrichment on sediment nitrogen removal in tidally influenced coastal systems. This study characterizes enrichment patterns in two tidal systems affected by wastewater treatment facility (WWTF) effluent and assesses the impact on habitat nitrogen removal via denitrification. We collected intact sediment cores from prevalent habitats in a tidal freshwater river (TFZ; swamp forest) and a tidal estuarine creek system (EST; salt marsh) upstream and downstream of a WWTF outfall, and quantified dissolved gas fluxes across the sediment‐water interface during wet conditions in early summer and dry conditions in late summer. Data collected during two synoptic water quality monitoring campaigns complimented laboratory experiments to provide environmental context for biogeochemical processing. The two systems exhibited different enrichment patterns such that the river‐dominated TFZ system was characterized by consistently elevated nitrate + nitrite concentrations downstream of the WWTF, whereas precipitation and tidal influence affected nutrient distributions in the EST creek. Downstream sediments in TFZ exhibit an apparent saturation response, while upstream rates may be limited by other factors, such as labile organic matter availability. In contrast, downstream sediments in EST denitrify at higher rates than upstream during wet conditions that may enhance transport of effluent. This work provides information on ecosystem functioning in human‐influenced environments and can be of use in developing nature‐based solutions, such as water treatment wetlands, for nitrogen removal.

## Core Ideas

- Enrichment patterns in tidal fresh and estuarine systems may affect sediment denitrification.
- A consistent downstream nitrate + nitrite (NO_x_) gradient in the tidal fresh river may explain denitrification ceiling.
- The estuarine creek experiences reversed gradients and elevated downstream denitrification.
- System‐wide denitrification rates were comparable in tidal fresh and estuarine environments.
- Implications for nature‐based solutions to remove nitrogen across a changing coastal landscape.

**Abbreviations**
DNF: denitrification
DS: downstream
EST: estuarine creek system
IMS: Institute of Marine Sciences
NOAA: National Oceanic and Atmospheric Administration
NO_x_
: nitrate + nitrite
NPDES: National Pollutant Discharge Elimination System
SOD: sediment oxygen demand
TFZ: tidal freshwater river
US: upstream
WWTF: wastewater treatment facility

## 1 INTRODUCTION

Eutrophication and water quality degradation in coastal environments are becoming more common as anthropogenic nitrogen loads increase (Malone & Newton, 2020; Maure et al., 2021). Coastal systems, such as wetlands, creeks, and estuaries can maintain water quality through the removal of nitrogen via denitrification (DNF) (Nixon et al., 1996; Piehler & Smyth, 2011; Velinsky et al., 2017). DNF is a microbial process found predominantly in anaerobic sediments that converts inorganic nitrogen (nitrate) to dinitrogen (N_2_) gas adding to the large pool of N_2_ in the atmosphere. DNF may account for a large portion of nitrogen removed in estuaries (Seitzinger, 1987) and can be a substantial sink for anthropogenic nutrients (Boustany et al., 1997; Pérez‐Villalona et al., 2015; Reisinger et al., 2016; Rosenzweig et al., 2018).

Studies have shown that coastal habitats can increase DNF capacities in response to elevated water column nitrate levels. Adame et al. (2019) even identified DNF “hotspots” in catchments characterized by high levels of nitrate. This response is habitat‐specific, often positively correlated with sediment organic matter (Smiley et al., 2023; Smyth et al., 2015; Tomasek et al., 2019). Results of these studies demonstrate ecosystem benefits for mitigating water quality degradation but are most representative of biogeochemical responses to a pulse of reactive nitrogen (e.g., during a storm) and less analogous to ecosystem functioning in chronically enriched systems.

Effects of chronic enrichment on nitrogen processing are variable. Semedo and Song (2019) observed reduced functional gene abundances and reduced DNF rates directly downstream (DS) of a wastewater treatment facility (WWTF) outfall. Other studies have suggested denitrifying communities are more prolific in nitrogen‐enriched waters compared with more pristine waters (Graves et al., 2016; Rahm et al., 2016) and that chronically enriched waters produce higher sediment DNF compared to relatively unpolluted, reference waters (García‐Ruiz et al., 1998; Lofton et al., 2007; Pattinson et al., 1998). These differences suggest that biogeochemical responses to chronic enrichment may be site‐specific. Strengthening our understanding of biogeochemical responses to chronic enrichment in coastal habitats will become increasingly important as development continues and anthropogenic nutrient loads increase.

Although many studies have shown that aquatic habitats respond to elevated nitrogen concentrations with higher nitrogen removal rates, limitations to DNF, especially in eutrophic systems, were recognized as early as the 1980s (Seitzinger & Nixon, 1985). Michaelis–Menten kinetics have been used to describe DNF in riverine systems (Evrard et al., 2012; García‐Ruiz et al., 1998; Ghane et al., 2015; Strong & Fillery, 2002; Yu et al., 2006), such that reaction rates approach a maximum (*V*_max_) and plateau with increased substrate concentrations, exhibiting a saturation response. *K*_m_ values (substrate concentration at one half the *V*_max_) reported in previous studies are highly variable, ranging between 1.5 and 7200 µM nitrate, dependent on localized availability of labile organic carbon, temperature, and soil oxidation‐reduction potential (Shiau et al., 2016; Strong & Fillery, 2002).Fewer studies have applied Michaelis–Menten kinetics to understand the impacts of nitrate enrichment on sediment biogeochemistry in tidal estuarine systems (Greene, 2005; Shiau et al., 2016). While rivers exhibit relatively predictable DS gradients of environmental variables (Vannote et al., 1980), tidal systems present complex and dynamic environmental variables. Tides intermittently transport nutrients, oxygen, and organic matter that likely influence nitrogen cycling (Lisa et al., 2017; Macias‐Tapia et al., 2021; Magni et al., 2002; Vörösmarty & Loder et al., 1994). Periodic inundation affects oxidation‐reduction potential in the sediments (Ensign et al., 2008; Grande et al., 2022; Knights et al., 2017), and abrupt fluctuations in temperature and salinity can affect biogeochemical processes (Marks et al., 2016; Rahman et al., 2019). An understanding of these processes is critical as sea levels rise and tidal influence extends further upstream (US).

The objectives of this study were to (1) characterize nitrogen enrichment patterns in a tidal freshwater river (TFZ) and an estuarine creek system (EST) considering tidal fluctuations and antecedent precipitation and (2) assess the effects of chronic nitrate enrichment on nitrogen processing by prevalent coastal habitats. This work specifically investigated enrichment via point source pollution from WWTFs. While regulatory measures and technological advancements have reduced nitrogen concentrations in wastewater effluent, point sources remain a significant contributor to nitrogen loads to waterways (Carey & Migliaccio, 2009; Finkler et al., 2023; Pieterse et al., 2003; Rajaei & Nazif, 2022), and loads will likely increase with population growth (Stow et al., 2001; Wang et al., 2019). Use of ambient habitats for wastewater treatment has been effective (Boets et al., 2021; Nichols, 1983); however, it is essential to understand factors that could enhance or inhibit their functioning for this purpose.

## 2 MATERIALS AND METHODS

### 2.1 Site description

**FIGURE 1:**
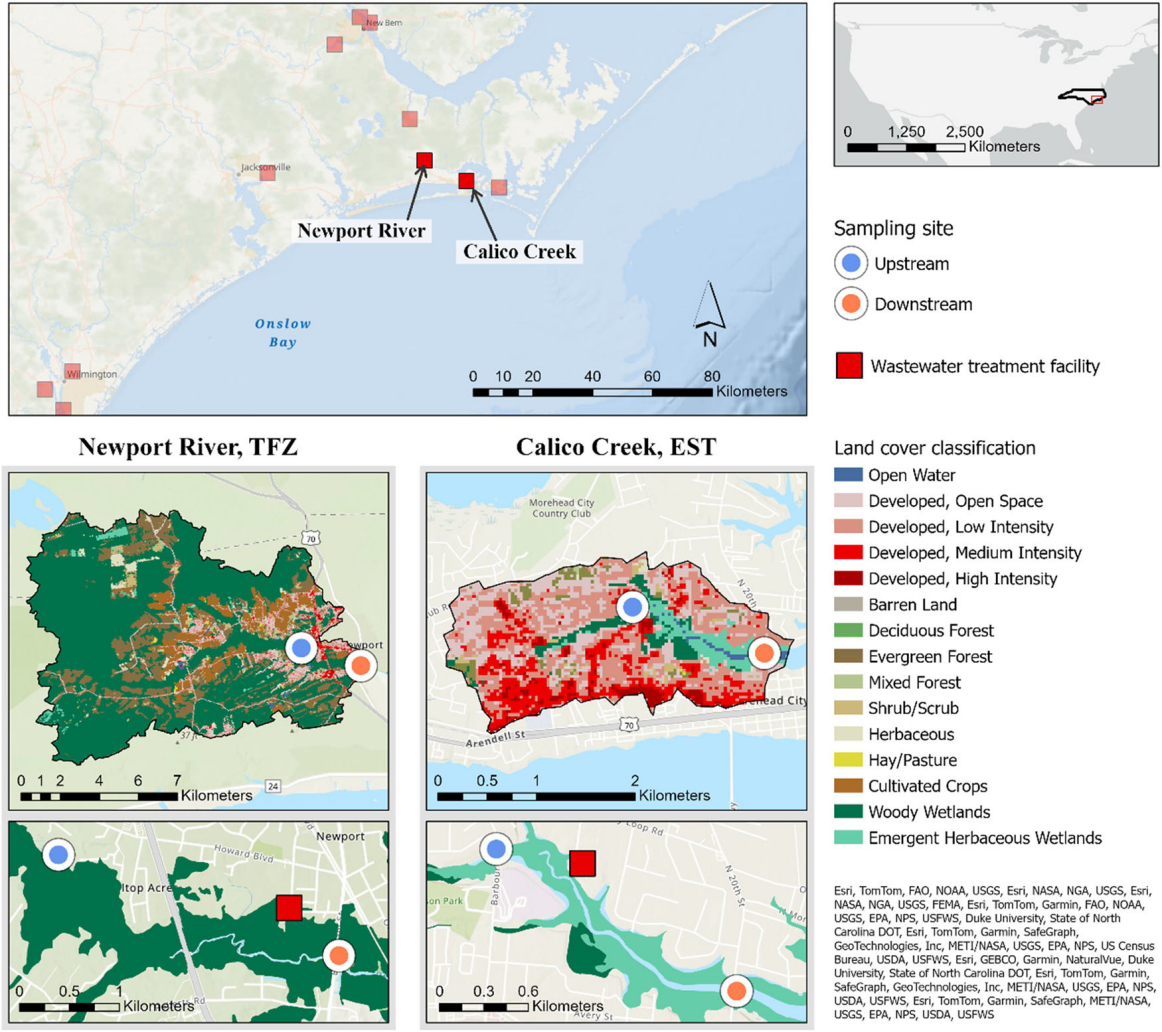
Map of coastal wastewater treatment facilities in eastern North Carolina, land cover within tidal freshwater river (TFZ) and EST watersheds, and prevalent wetland habitat at sediment core sampling sites. Land cover data were obtained from the National Land Cover Dataset 2019.

Site water and intact sediment cores (17 cm deep and 6.4 cm diameter) were collected from prevalent habitats in two tidal systems in Eastern North Carolina (Figure 1). These sites presented differences in tidal influence, prevalent habitat, and development intensity within the watershed. Cores were collected from habitats located US (*n* = 3) and DS (*n* = 3) relative to WWTF discharge points to investigate the influence of chronic nutrient enrichment on sediment nitrogen processing. Samples were collected in June and August 2021.

#### 2.1.1 Newport River, TFZ

The region of the Newport River sampled in this study is considered a tidal freshwater zone (TFZ), where salinities remain near zero parts per thousand and riparian areas are inundated at high tide, twice daily. Ensign et al. (2012) measured the tidal signal in the Newport TFZ, reporting maximum water levels of roughly 0.6 m above mean sea level 7 km US of the oligohaline estuary. The predominant riparian habitat in this region is swamp forest. The Newport River watershed is mostly undeveloped, with approximately 8.4% of the catchment classified as some level of developed. Behind forested habitats (woody wetland and evergreen forest), the next most abundant land cover category is cultivated crops, followed by developed open space and low‐intensity development. Ensign et al. (2007) also documented a nitrate + nitrite (NO_x_) concentration gradient that increased from the upper riverine boundary to the lower estuarine boundary of the Newport River system with a substantial contribution from the Newport WWTF to the total nitrogen load to the system (22%–100%). Sediment cores and water were collected at low tide in swamp forest habitat roughly 4.0 km US (TFZ‐US) and 0.7 km DS (TFZ‐DS) relative to the Newport Wastewater Treatment Plant (National Pollutant Discharge Elimination System [NPDES] Permit #NC0021555) in June and August of 2021. This relatively small municipal WWTF serves the Town of Newport and has a maximum treatment capacity of 1.2 million gallons per day.

**Core Ideas**

- Enrichment patterns in tidal fresh and estuarine systems may affect sediment denitrification.
- A consistent downstream nitrate + nitrite (NO_x_) gradient in the tidal fresh river may explain denitrification ceiling.
- The estuarine creek experiences reversed gradients and elevated downstream denitrification.
- System‐wide denitrification rates were comparable in tidal fresh and estuarine environments.
- Implications for nature‐based solutions to remove nitrogen across a changing coastal landscape.

#### 2.1.2 Calico Creek, EST

Calico Creek is a small tidal creek located in Carteret County, NC. This EST is characterized by semidiurnal tides that flood abundant salt marsh along the length of the creek and oyster reefs further DS. In contrast to the Newport watershed, the Calico Creek watershed is extremely developed with over 80% of the catchment characterized as developed. In compliance with the United States’ Federal Clean Water Act (1972; 33 U.S.C. §§ 1251 et seq.), the state of NC has compiled a 303(d) List of Impaired Water Bodies for which additional regulatory measures must be placed to meet a given water quality standard. A water body is considered nutrient impaired if chlorophyll‐a (chl‐a) concentrations are above 40 µg L^−1^. Calico Creek has been on the 303(d) List for nutrient impairment since 2008, with North Carolina's Division of Water Resources pointing to Morehead City's WWTF as a major source of water quality degradation since 1997 (Lin, 2020). Water and sediment samples were collected during low tide from salt marshes approximately 0.7 km US (EST‐US) and 1.4 km DS (EST‐DS) relative to the Morehead City Wastewater Treatment Plant (NPDES Permit #NC0026611). This municipal WWTF utilizes tertiary treatment methods and treats a maximum of 2.5 million gallons per day (Town of Morehead City Utilities Department, 2018).

### 2.2 Synoptic water quality sampling

Data collected during two separate monitoring campaigns provide environmental context for the biogeochemical processes measured in the laboratory. The first was a year‐long monitoring effort along a tidal freshwater transect in the Newport River from 2006 to 2007 (Ensign et al., 2007). TFZ‐US and TFZ‐DS were among the sites sampled. The second monitoring campaign lasted 17 months from 2021 to 2022, where samples were collected in the Newport Estuary and Calico Creek, including the EST‐US and EST‐DS sites. Both monitoring efforts involved collecting water samples one to two times monthly. In situ measurements included salinity, temperature, dissolved oxygen, and chl‐a concentrations. Water samples were brought to the University of North Carolina's Institute of Marine Sciences (IMS) in Morehead City, NC, to measure concentrations of dissolved NO_x_ and chl‐a. The water samples were filtered through 0.7 µm Whatman GF/F filters and stored at −18°C prior to analysis. Concentrations of nutrient analytes, including NO_x_, were quantified using a Lachat Quick‐chem 8000 (Lachat Instruments, Milwaukee). Filters were analyzed for chl‐a. Note that chl‐a was extracted from frozen filters in a 90% acetone solution, and fluorescence of the extract solution was measured using a Turner Designs Trilogy fluorometer (Welschmeyer, 1994).

These water quality data were paired with precipitation data obtained from the NC Climate Office for the Beaufort Airport (station KMRH; https://econet.climate.ncsu.edu/m/?id = KMRH). Precipitation data were available at 1‐h intervals and were used to calculate total rainfall in the 72 h preceding water quality sampling. These data were corroborated using the Community Collaborative Rain, Hail & Snow Network citizen science rain gauge measurements (https://www.cocorahs.org/ViewData/ListDailyPrecipReports.aspx). When precipitation amounts exceeded 0.5 in. within the 72‐h window, conditions were considered “wet.” If it rained <0.5 in. during this period, conditions were considered “dry.” Additionally, water level data at EST were obtained from the National Oceanic and Atmospheric Administration (NOAA) tide gauge at Beaufort, NC (Station ID 8656483; https://tidesandcurrents.noaa.gov/waterlevels.html?id = 8656483). This tide gauge is approximately 6 km from Morehead City's WWTF and experiences similar tidal effects. Tidal ranges were considered “high” when the average high water level (average of the two recorded high tides) was within the fourth quartile for the monitoring period.

### 2.3 Gas and nutrient flux measurements

Flux measurements of dissolved gases (N_2_ and O_2_) across the sediment‐water interface of sediment cores were obtained during June and August 2021 using methods described by Piehler and Smyth (2011). Swamp forest (TFZ) and salt marsh (EST) sediment cores and site water were transported to IMS. The cores were maintained submerged in the dark in a temperature‐controlled environmental chamber (Bally Inc.) set to in situ temperatures. During core incubation, site water was continuously aerated in feedwater bins and was pulled through the overlying water column of corresponding sediment cores at a rate of 0.6 L h^−1^ for a roughly 5‐h turnover. Following an overnight equilibration period, feedwater inflow and outflow water samples from each core were collected at three timepoints 5 h apart.

A membrane inlet mass spectrometer (Bay Instruments) was used to measure ratios of dissolved gases, N_2_:Ar and O_2_:Ar, immediately following each water sample collection. Extra inflow and outflow samples (approximately 40 mL) were collected at the second timepoint to measure NO_x_ concentrations using the methods described above.

### 2.4 Calculations and statistical analysis

Water quality monitoring data were used to investigate spatiotemporal variations in nutrient concentrations in the TFZ and EST systems as well as the effects of water levels and antecedent precipitation on enrichment. First, system NO_x_ concentrations and chl‐a during wet and dry conditions were compared by combining US and DS measurements in TFZ and EST. Then, to characterize enrichment patterns in each system, US NO_x_ concentrations were subtracted from DS. Positive differences were classified as “standard gradients,” where DS water was nutrient enriched relative to US. Conversely, negative differences were considered “reversed gradients,” where concentrations US were higher than DS. These concentration differences were then grouped based on environmental conditions. The TFZ data were grouped within “wet” and “dry” conditions, and the EST data were grouped within “wet,” “dry,” and “dry‐high water” (dry‐HW) conditions (*n* = 9, 19, 4, respectively). Tide gage data were not available for the TFZ system; therefore, a dry‐HW scenario was not considered for that system. Furthermore, dry‐HW conditions were associated with significant fluctuations in NO_x_ concentrations (e.g., periodic reversed gradients) in EST that were not observed in TFZ. These data did not fulfill normality assumptions (Shapiro–Wilk test; *p* < 0.05). Thus, mean ranks across environmental conditions were compared using Kruskal–Wallis and post hoc Dunn tests.

Nutrient and dissolved gas flux rates were calculated by comparing concentrations in outflow samples to inflow samples using the following equation:

$$\mathrm{Flux}=\frac{\left(C_{\mathrm{outflow}}-C_{\mathrm{inflow}}\right)\;\times \;F}{A}$$

where *C* represents concentration, *F* represents the peristaltic pump/flow rate, and *A* represents the sediment surface area within the core. Means for each system were compared across environmental conditions and location relative to a WWTF using Kruskal–Wallis and post hoc Dunn tests. Linear regressions were used to assess the relationships between DNF and sediment oxygen demand (SOD; O_2_ flux multiplied by negative one) during wet and dry conditions. All statistical tests were conducted using R version 4.1.1 and were considered significant when *p* < 0.05.

## 3 RESULTS

### 3.1 Environmental conditions and enrichment patterns in TFZ and EST

Synoptic water quality monitoring data from US and DS sites were grouped for both the TFZ and EST to assess the effects of antecedent precipitation on system NO_x_ and chl‐a concentrations (Table 1). In TFZ, there was no significant rainfall effect on NO_x_ or chl‐a concentrations. In EST, NO_x_ concentrations were significantly higher during dry periods than wet, and there were no significant differences between chl‐a concentrations.

**TABLE 1:** Summary of system‐wide (combined upstream and downstream measurements) water quality measurements for the tidal freshwater river (TFZ) and estuarine creek system (EST) during wet and dry periods.

Sampling site	Treatment	N	NO_x_ (µM)	Chl‐a (mg $L^{-1}$)
TFZ	Wet	19	9.39 ± 2.45	3.97 ± 1.09
	Dry	30	10.0 ± 1.75	4.06 ± 1.26
EST	Wet	18	17.6 ± 5.68^a^	21.0 ± 6.19
	Dry	34	28.1 ± 4.00^b^	35.1 ± 8.28
	Dry‐HW	12	24.3 ± 9.29^a,b^	10.2 ± 3.88

*Note*: Data are reported as mean value ± standard error. Superscript letters denote statistically significant differences.

Abbreviations: Chl‐a, chlorophyll‐a; Dry‐HW, dry‐high water; NO_x_, nitrate + nitrite.

**FIGURE 2:**
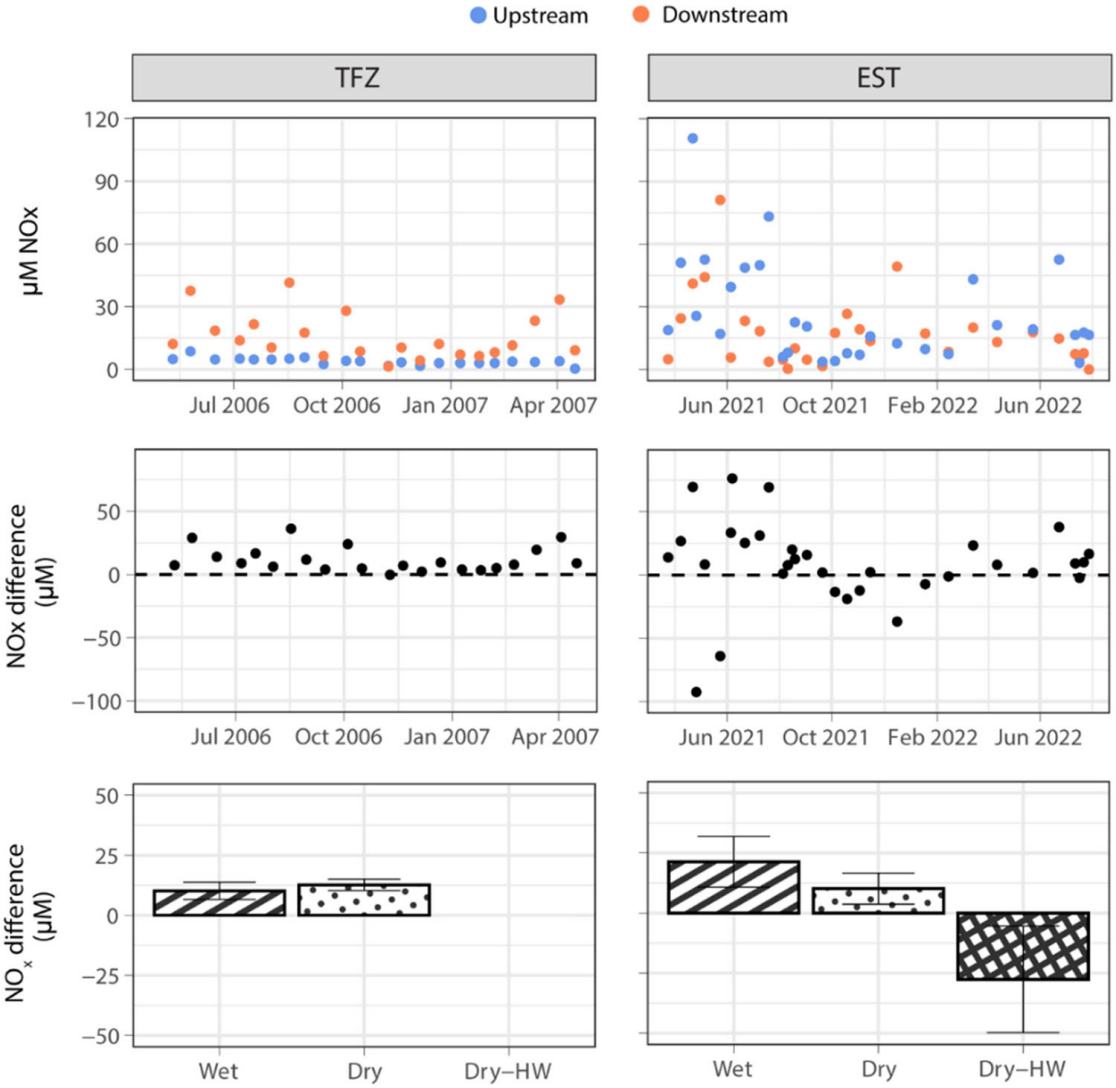
Ambient nitrate + nitrite (NO_x_) concentrations upstream and downstream relative to wastewater treatment facility (WWTF) outfall in both tidal freshwater river (TFZ) and EST systems (top), differences between upstream and downstream concentrations (middle), and mean concentration differences grouped by conditions.

In TFZ, NO_x_ concentrations ranged from 0.360 to 8.57 µM at the US site and 1.40 to 41.6 µM at the DS site. DS NO_x_ concentrations were consistently and significantly higher than US under both wet and dry conditions. After grouping concentration differences based on antecedent precipitation, there was no significant difference between wet and dry means (10.2 ± 3.61 and 12.7 ± 2.36 µM, respectively; mean value ± standard error; Figure 2). All monitoring data are included in Table S1, and ambient water quality measurements on sediment core collection dates are included in Table S2.

**FIGURE 3:**
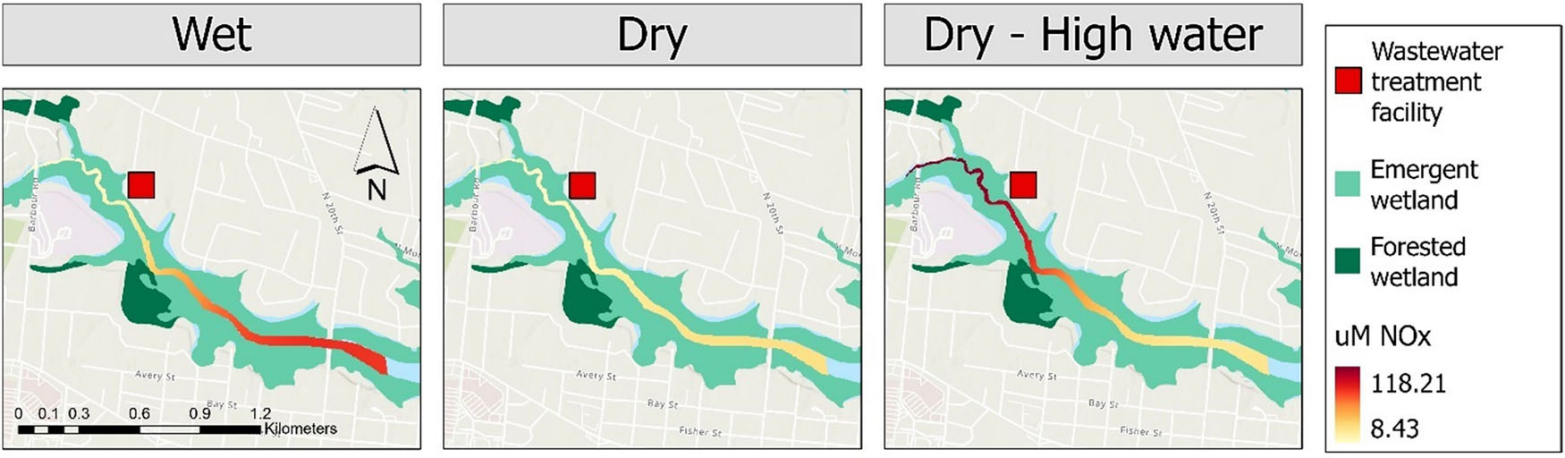
Nitrate + nitrite (NO_x_) concentration gradient in estuarine creek system (EST) during wet (June 6, 2021), dry (August 17, 2021), and dry‐HW (April 26, 2021) conditions. Map credits: Esri. NASA, NGA, USGS, FEMA, State of North Carolina DOT, HERE, Garmin, SafeGraph, GeoTechnologies, Inc., METI/NASA, EPA, NPS, US Census Bureau, USDA.

EST NO_x_ concentrations were more variable than TFZ, with periodic reversed gradients. At EST‐US, concentrations ranged from 0.0300 to 118 µM. At EST‐DS, concentrations ranged from 3.21 to 110 µM. The mean concentration difference produced during dry‐HW tidal conditions was −27.6 ± 22.2 µM (Figure 2), which was significantly less than means produced during wet conditions. During wet and dry periods (standard water levels), DS NO_x_ levels were significantly higher than US, translating to positive concentration differences at standard tidal regimes (Figure 2; 21.3 ± 10.5 and 10.2 ± 6.50 µM, respectively). Figure 3 illustrates strong standard gradients produced during wet conditions, a relatively weaker standard gradient under dry conditions, and a reverse gradient during dry‐HW conditions.

Over the course of the 2007 monitoring campaign, chl‐a concentrations in TFZ ranged from 0.80 to 26.1 mg L^−1^. Concentrations were significantly higher DS compared to US during wet periods. Salinity in TFZ was fresh, ranging from 0.030 to 0.20 ppt with no significant differences between US and DS sites or between wet and dry conditions. Chl‐a and salinity were higher and more variable in EST and exhibited patterns with precipitation and water levels. Mean chl‐a concentrations were higher at EST‐DS (27.3 ± 10.6 mg L^−1^) than EST‐US (9.95 ± 1.88 mg L^−1^) during wet conditions, though not statistically significant. The opposite was observed during dry and dry‐HW conditions, where chl‐a concentrations were higher US than DS. The highest chl‐a concentrations were observed at EST‐US during dry conditions with a mean of 73.3 ± 16.2 mg L^−1^, significantly higher than DS concentrations of 18.4 ± 6.16 mg L^−1^). During dry‐HW conditions, chl‐a concentrations were 21.9 ± 9.18 mg L^−1^ at EST‐US and 6.28 ± 3.61 mg L^−1^ at EST‐DS (*p* = 0.07). Salinity ranged from 0 to 33.3 ppt.

### 3.2 Denitrification in TFZ and EST

**FIGURE 4:**
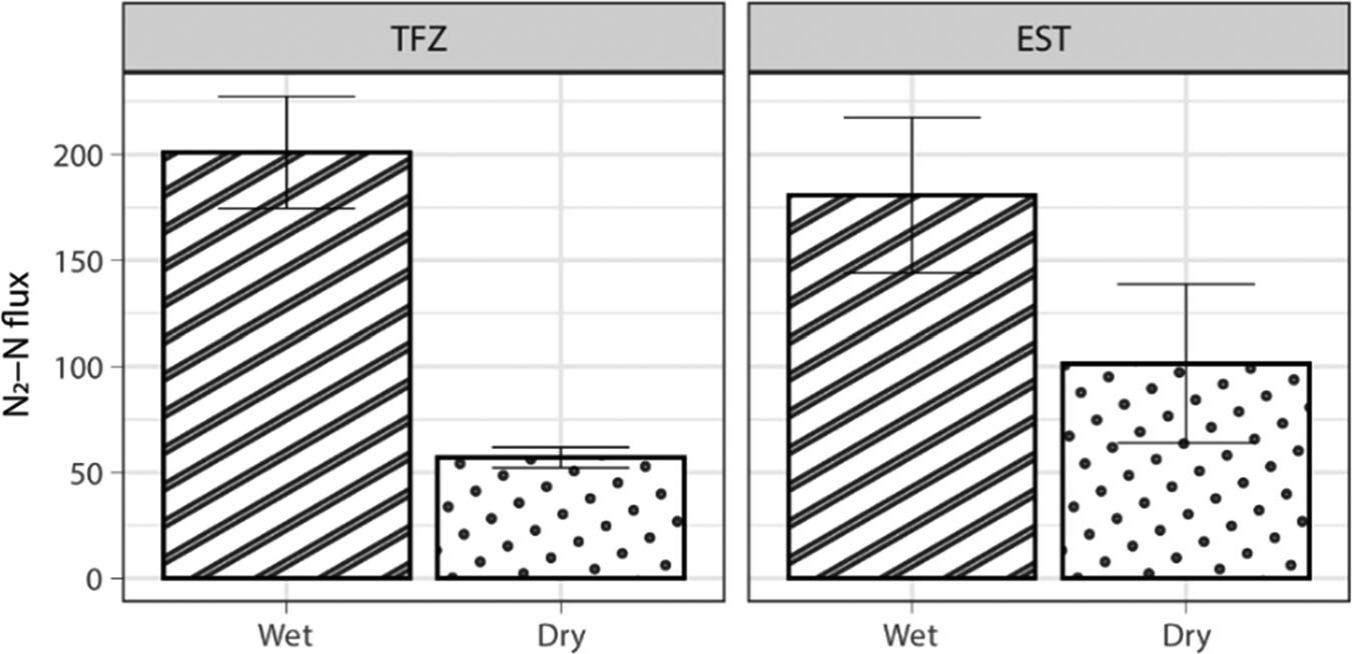
System‐wide N_2_‐N flux measurements (µmol m^−2^ s^−1^; upstream and downstream measurements combined) on wet and dry sampling dates in tidal freshwater river (TFZ) and estuarine creek systems (EST).

To compare nitrogen processes in the swamp forest sediments of the TFZ system to the salt marsh sediments of the EST system, rates measured at US and DS sites within each creek were combined (Figure 4). N_2_‐N fluxes during wet conditions were comparable between TFZ and EST samples (201 ± 56.3 µmol m^−2^s^−1^ and 181 ± 46.2 µmol m^−2^s^−1^, respectively). The same was true during dry conditions (TFZ, 57.1 ± 7.37 µmol m^−2^ s^−1^; EST, 101 ± 46.2 µmol m^−2^s^−1^). In both systems, mean DNF rates were significantly higher during wet conditions compared to dry. All N_2_‐N flux measurements are included in Table S3.

### 3.3 Enrichment effects on nitrogen processing

**FIGURE 5:**
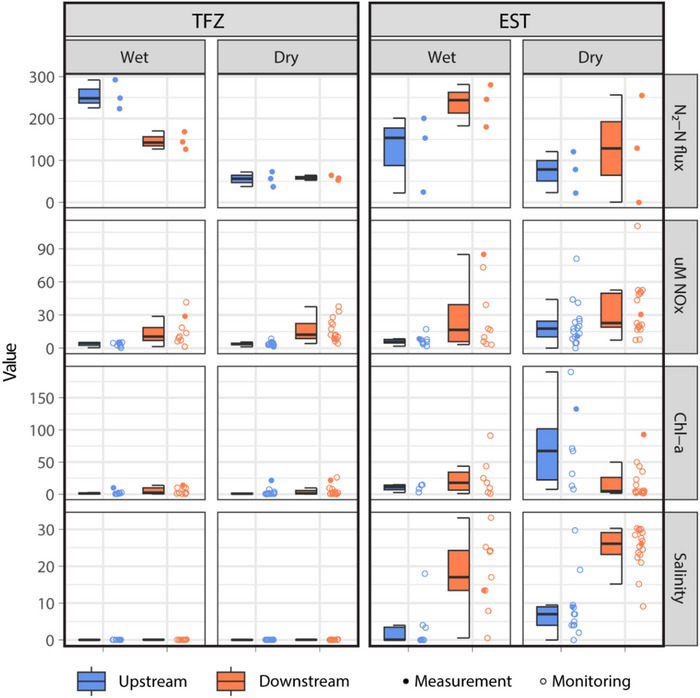
Upstream and downstream N2‐N flux measurements (µmol m^−2^ s^−1^), as well as nitrate + nitrite (NO_x_) concentrations (µM), chlorophyll‐a (chl‐a) levels (mg L^−1^), and salinity (ppt) in the tidal freshwater river (TFZ) and estuarine creek system (EST) on wet and dry sampling dates during water quality monitoring campaigns. Solid points correspond with data from core collection.

Water quality data were collected from the TFZ system approximately 15 years prior to the flux measurements collected in this study. Nitrate, chl‐a, and salinity measurements from core collection dates fell within the ranges established in the monitoring data, and the DNF rates measured in this study were strikingly similar to those measured by Ensign et al. (2008) and Von Korff et al. (2014). Furthermore, a comparison of NOAA Coastal Change Analysis Program (C‐CAP) land cover data from 2006 and 2016 (https://coast.noaa.gov/digitalcoast/data/ccapregional.html) revealed minimal changes in total areas of wetlands, forests/scrublands, agricultural lands, and developed areas within the TFZ watershed (Figure 1). It was assumed that these land cover changes had negligible effects on water quality and quantity, and data collected in 2006–2007 from the TFZ were representative of environmental conditions on core collection dates. In the TFZ system, synoptic water quality sampling data revealed chronic enrichment DS of the WWTF relative to US. DNF rates were higher in US swamp forest sediments (255 ± 19.5 µmol m^−2^s^−1^) compared to DS (147 ± 12.7 µmol m^−2^s^−1^) on the wet sampling date, although this relationship was not statistically significant (Figure 5). TFZ‐US and TFZ‐DS DNF rates were comparable during dry conditions (55.6 ± 10.1 µmol m^−2^s^−1^ and 58.6 ± 3.43 µmol m^−2^s^−1^, respectively).

The EST system experienced nitrogen enrichment DS of the WWTF compared to US but was also periodically characterized by reversed NO_x_ concentration gradients over the course of the monitoring period. The strongest standard gradients occurred during wet conditions, and there was an observed effect on DNF (Figure 5). During wet conditions, EST‐DS sediment cores exhibited significantly higher N_2_‐N fluxes (236 ± 28.8 µmol m^−2^s^−1^) than EST‐US cores (126 ± 53.4 µmol m^−2^s^−1^). US and DS DNF rates were not significantly different during dry conditions (74.2 ± 28.3 and 128 ± 73. m^−2^s^−1^, respectively).

**FIGURE 6:**
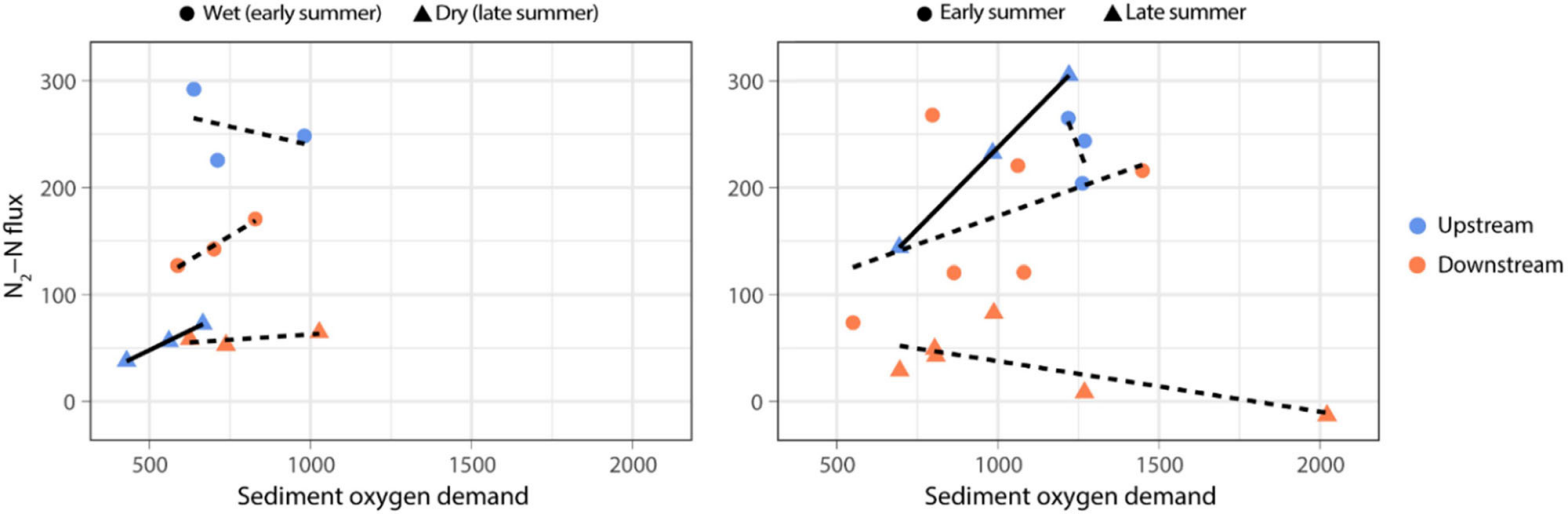
Linear regressions comparing relationships between sediment oxygen demand and denitrification in tidal freshwater river (TFZ). Panel (A, left) includes data from the wet and dry core collection dates. Panel (B, right) includes data from previous studies in the TFZ, including Von Korff et al. (2014; upstream sites) and Ensign et al. (2008; downstream sites). Solid line indicates statistically significant linear relationship (*p* < 0.05).

SOD was variable, and no significant differences were detected between US and DS cores or between wet and dry conditions in either system. Linear regressions were used to assess the relationships between N_2_‐N fluxes and SOD on the wet and dry sampling dates in TFZ only (Figure 6) since no significant relationships for these variables were evident in EST. SOD is an indicator of aerobic activity in the sediments, including nitrification, and its relationship with N_2_‐N flux has been used to determine the relative importance of coupled nitrification‐DNF (Fennel et al., 2008; Piehler & Smyth, 2011). The only significant relationship was at the TFZ‐US site during dry conditions (Figure 6A), but seasonal patterns show some alignment with previous data at TFZ‐US and TFZ‐DS (Figure 6B), for which significance was also demonstrated for regressions between SOD and N_2_‐N flux at US sediments during early summer. Slopes, *R* ^2^ values, and *p* values are summarized in Table S4.

## 4 DISCUSSION

### 4.1 Enrichment patterns in TFZ and EST

Tidal excursions affect distribution of materials and physicochemical conditions that can in turn affect biogeochemical cycling (Grande et al., 2022; Lisa et al., 2017; Macias‐Tapia et al., 2021; Magni et al., 2002; Vörösmarty & Loder et al., 1994). Therefore, before assessing the effects of chronic enrichment on DNF, enrichment patterns in the TFZ and EST systems were established considering both antecedent precipitation and tidal influence.

TFZ and EST sites exhibited different enrichment patterns. DS waters in the TFZ system were consistently NO_x_ enriched compared to US waters, and there was no apparent rainfall effect on the degree of the enrichment over the course of the synoptic sampling period. These results suggest that riverine forcing primarily dictates nutrient distributions and export in this system, and tides have lesser influence. This dominant riverine forcing has been documented in other tidal fresh systems (Devore, 2016; Stern et al., 1991), making them hydrodynamically more analogous to riverine systems in which Michaelis–Menten reaction kinetics have been used to describe sediment nitrogen processing (Evrard et al., 2012; García‐Ruiz et al., 1998; Ghane et al., 2015; Strong & Fillery, 2002).

Enrichment in the EST system was more variable compared to the TFZ system. NO_x_ concentration gradients were affected by both rainfall and tides. Standard concentration gradients for NO_x_ were documented during wet and dry periods but were most pronounced when it had rained over 0.5 in. in the previous 72 h. Increased discharge following rainfall likely flushes materials, including NO_x_ and DS. Reversed gradients were observed in EST during dry conditions and tidal excursions of higher amplitude (within the fourth quartile for the monitoring period). Reversed nutrient gradients were recorded in Calico Creek during a 2018 monitoring effort conducted by the North Carolina Division of Water Resources (Modeling and Assessment Branch, 2021), and Mallin et al. (2004) observed reversed gradients in urban tidal creeks in New Hanover County, NC. Although the relationships between those occurrences and rainfall were not reported in either study. A 2006 study conducted in the Florida Everglades assessed seasonal salinity–nutrient relationships and described an “upside down estuary” during the dry season, where reduced freshwater inflow results in the marine end‐member of the estuary serving as the source of limiting nutrients (Childers et al., 2006). Although the Everglades study focused on seasonal fluctuations in rainfall, the relationship between precipitation and tidal influence may be applicable in the EST system.

Tidal fluctuations on the shorter timescale have variable effects on nutrient concentrations. Some studies have reported reduced NO_x_ concentrations during spring tides compared to neap tides (Moser et al., 2005) and flood tides compared to ebb tides (Magni et al., 2002; Vörösmarty & Loder et al., 1994), likely due to dilution effects with increased water volumes. Additional studies, however, have shown that episodic high water events, like king tides, produce high concentrations of dissolved inorganic nitrogen in the water column by mobilizing land‐bound nutrients (Macias‐Tapia et al., 2021; Macías‐Tapia et al., 2023; Mulholland et al., 2022). This could be what was observed in the EST system during dry‐HW periods. Furthermore, it is evident that US tidal delivery of WWTF nutrients is most pronounced when freshwater inputs are low, as evidenced by reversed gradients observed in EST during dry‐HW conditions, a phenomenon that has not previously been documented.

### 4.2 Precipitation and seasonal effects on nitrogen processing

Despite differences in enrichment patterns, swamp forest sediments in TFZ and marsh sediments in EST exhibit net DNF at comparable rates. TFZ swamp forest sediments and EST marsh sediments exhibited higher DNF rates on the wet sampling date than the dry sampling date. It is possible that increased soil moisture following rain events decreased sediment oxidation‐reduction potential and favors DNF, as observed by Ensign et al. (2008) in the Newport TFZ in 2007. Increased nitrate availability for direct DNF following a rain event in June 2021 may also explain elevated N_2_‐N fluxes (Smiley et al., 2023). Water column NO_x_ concentrations were four times higher on the wet core collection date than the dry core collection date, both US and DS in TFZ and nearly three times higher at EST‐DS, likely from increased non‐point source inputs via stormwater runoff as well as DS delivery of effluent from the treatment plants. Additionally, chl‐a concentrations on the wet sampling date were approximately half of those measured on the dry sampling date at both TFZ‐US and TFZ‐DS and one‐tenth of those at EST‐DS. The inverse relationship between NO_x_ concentrations and chl‐a in both systems could be attributed to longer residence times that favor primary productivity and nutrient uptake from the water column (Childers et al., 2006) that could present competition with sediment denitrifiers for NO_x_.

Interestingly, elevated NO_x_ concentrations measured on core collection dates do not follow trends established in the monitoring data. For example, in EST, system‐wide monitoring trends showed NO_x_ concentrations were higher during dry periods than wet in this tidal system. This may be an artifact of the study design, comparing measurements from select dates to long‐term trends. The difference also reflects the complex concentration‐discharge relationships in urban estuaries. Gold et al. (2019) observed hemodynamic export of NO_x_ as well as dilution in catchments characterized by high levels of imperviousness, similar to EST. While stormwater can deliver additional nutrients to receiving waterways, increased water volume in the creek can also dilute nutrients from point sources, with these concentration‐flow relationships further confounded by tidal exchange. EST chi‐*a* concentrations on core collection dates did follow trends established by the monitoring data such that concentrations were higher during dry periods than wet, which supports the idea that higher residence times favor primary productivity.

In the TFZ system, seasonal variables in addition to rainfall likely explain lower DNF rates as the summer progresses—the wet sampling date was in early June and the dry sampling date fell in mid‐August. Earlier research by Ensign et al. (2008) in swamp forest sediments proximal to the TFZ‐DS site reported DNF rates of 199 and 52.5 µmol m^−2^s^−1^ in June and August 2007, respectively, concurring with seasonal patterns currently observed. Greene (2005) discussed predictable decreases in water column NO_x_ concentrations and an increase in relative importance of coupled nitrification‐DNF as the summer progressed in the Patuxent tidal freshwater system. Similarly, the Newport TFZ system exhibited lower NO_x_ concentrations in August than in June at both US and DS sites and a significant linear relationship between SOD and N_2_‐N fluxes in TFZ‐US sediments in August, providing evidence for coupled nitrification‐DNF that was not observed earlier in the summer. Annual gas flux data from Von Korff et al. (2014) in TFZ‐US sediments showed a significant linear relationship between SOD and N_2_‐N flux, indicative of nitrification‐DNF coupling that was most pronounced in the late summer. In contrast, swamp forest sediments DS of TFZ WWTF showed no relationship between SOD and N_2_‐N flux in the late summer when DNF rates were lower, which concurred with patterns that Ensign et al. (2008) observed during August and September in these sediments. It is possible that as the summer progresses, depletion of labile organic matter via heterotrophic remineralization is directly limiting DNF, particularly at US location. In DS sediments, shifts in sediment oxidation‐reduction potentials as sediments become anoxic likely limit nitrification‐DNF coupling and could shift direct nitrate processing to alternate pathways such as dissimilatory nitrate reduction. This could suggest WWTF as a source for labile organic matter to DS sediments (Krasner et al., 2009; Shon et al., 2006). The seasonal effect on DNF in the TFZ system that was not evident in the EST systems mimics the findings reported by Neubauer et al. (2005), that hydrology (e.g., water table depth) had a stronger effect on biogeochemical processes in a brackish marsh, whereas plant‐mediated, or seasonal, processes have a stronger effect in tidal fresh marsh sediments.

### 4.3 Enrichment effects on nitrogen processing

In the TFZ system, US DNF rates were higher than DS during wet conditions; in contrast to EST, where DS DNF rates were higher than US during both dry and wet conditions, with a significant US‐DS difference during wet conditions. Nitrogen enrichment patterns in these two systems appear to be responsive to hydrologic factors that result in different observed patterns in DNF.

Michaelis–Menten kinetics have been used to describe DNF in riverine systems such that a maximum reaction rate exists independent of increased substrate (e.g., NO_x_) concentrations (Evrard et al., 2012; García‐Ruiz et al., 1998; Ghane et al., 2015; Strong & Fillery, 2002). In the TFZ system, where riverine forcing is the primary driver of nutrient distributions evidenced by a consistent standard NO_x_ concentration gradient, the DS sediments may have reached a maximum processing threshold despite elevated NO_x_ concentrations and SOD that would plausibly favor direct DNF. Though our study design did not include amending nitrate concentrations to assess a DNF response, saturation responses were previously suggested in this TFZ system (Von Korff et al., 2014). In an analogous tidal freshwater system, the Patuxent River, Greene (2005) reported a maximum DNF rate (*V*_max_) of 238 µM N m^−2^h^−1^ and a *K*_m_ value (substrate concentration at ½ *V*_max_) of 93 µM nitrate in marsh sediments. This DNF ceiling is lower than rates measured in TFZ swamp forest sediments by Ensign et al. (2008) and Von Korff et al. (2014) but supports an interpretation that chronically nitrate‐enriched DS TFZ sediments may not have the capacity to increase DNF rates in response to elevated NO_x_ concentrations.

In EST, it is possible that more dynamic tidal exchange results in frequent fluctuation of nitrate concentrations that interfere with saturation kinetics and favor opportunistic direct DNF. Shiau et al. (2016) used Michaelis–Menten kinetics to describe DNF response to elevated nitrate concentrations in Southeast Asian mangrove sediments along a river‐estuary gradient. While they report DNF rates and NOx concentrations orders of magnitude higher than those recorded in the TFZ and EST systems, they found an US, low salinity site yielded significantly lower *V*_max_ and *K*_m_ values than DS higher salinity sites. This suggests that the DNF ceiling in tidal freshwater systems may be lower than in estuarine systems further DS, as observed in this study.

### 4.4 Landscape context and nitrogen processing

Differences in N_2_‐N fluxes and responses to chronic enrichment from WWTF effluent may reflect differences in land cover as well. The region of the TFZ sampled in this study drains a relatively undeveloped watershed and is characterized by predominant swamp forest, high levels of colored dissolved organic matter, and low irradiance (Ensign et al., 2012). In contrast, the EST system drains an urban watershed and is characterized primarily by salt marsh and abundant sunlight. Land cover composition within both watersheds is illustrated in Figure 1.

Development within a watershed can influence concentrations of nitrogen entering a system. Increased population densities and abundance of impervious surfaces can increase the overall amount of nitrogen entering the system as well as expedite delivery of nitrogen to the system (Bernhardt et al., 2008). Greater anthropogenic non‐point source inputs could explain the relatively high NO_x_ concentrations measured in EST compared to TFZ. Elevated NO_x_ concentrations may also explain why DNF rates are comparable in the two systems despite high salinities and relatively low SOM in the tidal estuary (Table S1), which have been linked to reduced DNF rates compared to freshwater habitats in previous studies (Craft et al., 2009; Marks et al., 2016; Pan et al., 2023).

EST's highly developed watershed contrasts the TFZ's forest‐dominated watershed. The TFZ‐US site receives input primarily from forested wetlands, and prevalence and intensity of developed landscapes appear to increase DS near WWTF. Thus, the relative human contribution to total nitrogen loads likely differs between TFZ‐US and TFZ‐DS, such that both point sources (e.g., WWTF effluent) and non‐point sources (e.g., urban runoff) markedly increase human contributions at the DS site. Although the relationship was not statistically significant, it is notable that swamp forest DNF rates were higher at the US site compared to DS. This may reflect a shift in primary nitrogen source from terrestrial US to human sources DS and suggests that nitrogen removal by riparian buffers is more effective in more pristine environmental contexts.

Palustrine swamp forests are expected to convert to emergent wetland marshes as sea levels rise. Simultaneously, coastal population growth and urban expansion are expected to continue. Therefore, understanding the land use context and impacts on ecosystem functioning will be increasingly important.

## 5 CONCLUSIONS

This work characterized nitrate enrichment patterns and assessed the effects on sediment nitrogen processing in prevalent wetland habitats of two tidal systems. Water treatment wetlands are common nature‐based solutions, and these results showed that environmental context is important for nitrogen removal efficacy.

The dominant riverine influence in the TFZ contrasted with the tidal influence in the EST. Differences in enrichment patterns likely affect biogeochemical response to chronic exposure to WWTF effluent. TFZ exhibited a consistent NO_x_ concentration gradient, and DS DNF rates may reach an upper nitrate processing threshold while US rates appear to respond to other limiting factors, such as seasonal fluctuations in organic matter availability. In contrast, elevated DNF rates in EST‐DS sediments may reflect dynamic tidal flushing and fluctuations in NO_x_ concentrations that lead to opportunistic direct DNF along with coupled nitrification‐denitrification. Furthermore, these findings have implications for water quality as regional population growth is often accompanied with a transition from septic to sewer systems, increasing demand on WWTFs. When designing treatment wetlands, integrating or mimicking tidal exchange could help to maximize nitrogen removal potential.

High DNF rates in US TFZ sediments compared to DS were notable and suggest that landscape context is important for nitrogen removal. It is plausible that riparian buffers are most effective in more pristine environments. Riparian buffer conservation is a common management practice for regulating water quality; however, their nitrogen removal functioning may be negatively affected by development within the watershed.

In the longer term, as sea levels rise, estuarine marshes and palustrine forests will migrate to higher elevations. Inland development may prevent this migration and reduce the landscape's nitrogen removal capacity. In the coastal regions, swamp forests are expected to transition to estuarine marshes, and there may be opportunities to conserve water quality regulation functions. EST marsh sediments produced comparable system‐wide DNF rates to TFZ swamp forest sediments, with DS marsh sediments producing higher DNF rates with elevated NO_x_ concentrations from the WWTF. Continuing to protect riparian buffers and preserve potential migration corridors will be essential for the resilience of coastal ecosystems and maintaining ecosystem function.

Results of this work improve our understanding of water quality regulation capacity by coastal habitats in human‐influenced environments. As population growth in coastal regions continues, the demand on WWTFs will increase, as will anthropogenic nitrogen loads to waterways. Furthermore, wetlands are a critical natural feature and a common component of nature‐based solutions; thus, understanding human and environmental factors that may enhance or inhibit their functioning in a changing climate is essential.

## AUTHOR CONTRIBUTIONS

**Anne Margaret H. Smiley**: Conceptualization; data curation; formal analysis; investigation; visualization; writing—original draft; writing—review and editing. **Suzanne P. Thompson**: Conceptualization; investigation; writing—review and editing. **Michael F. Piehler**: Conceptualization; funding acquisition; writing—review and editing.

## CONFLICT OF INTEREST STATEMENT

The authors declare no conflicts of interest.

## Supporting information



Supplemental materials include tables that water quality measurements from synoptic sampling campaigns, ambient conditions on sediment core collection dates, N_2_‐N flux values, and products of linear regression analyses.

Supporting Information

Supporting Information

## Data Availability

Data collected during water quality monitoring campaigns and sediment core incubation measurements are available upon request.
