# Supplementary material for: Chronic enrichment affects nitrogen removal in tidal freshwater river and estuarine creek sediments
Source: J Environ Qual. 2025 Jan 13;54(2):420–34. doi: 10.1002/jeq2.20674 (PMC11893282; doi:10.1002/jeq2.20674)
Supplement: Supplementary file 1 — Supplemental materials include tables that water quality measurements from synoptic sampling campaigns, ambient conditions on sediment core collection dates, N2‐N flux values, and products of linear regression analyses. [file JEQ2-54-420-s001.docx]

Supplemental Material

*Supplementary Table S1. Monitoring data collected from the 2006-2007 and 2021-2022 monitoring campaigns. See excel file.*

| *Supplementary Table S2. Summary of ambient conditions on sediment core collection dates. *Temperature, salinity, dissolved oxygen, and chl-a measurements for EST during wet conditions are from 6/5/2021.* | | | | | | | |
| --- | --- | --- | --- | --- | --- | --- | --- |
| **Treatment** | **Sampling site** | | **Temp** | **Salinity** | **ODO** | **Chl-a** | **[NOx]** |
|  |  | | **֯C** | **ppt** | **mg L^-1^** | **µg L^-1^** | **µM** |
| Wet  *6/7/2021** | TFZ | Upstream | 23.1 | 0.070 | 5.63 | 10.09 | 5.20 |
|  |  | Downstream | 25.1 | 0.100 | 4.33 | 13.88 | 28.9 |
|  | EST | Upstream | 26.0 | 0.00 |  | 13.99 | 8.43 |
|  |  | Downstream | 26.4 | 13.4 | 3.64 | 6.16 | 84.9 |
| Dry  *8/17/2021* | TFZ | Upstream | 25.7 | 0.040 | 4.94 | 21.5 | 1.14 |
|  |  | Downstream | 26.3 | 0.040 | 4.24 | 21.6 | 8.64 |
|  | EST | Upstream | 27.1 | 3.84 | 2.41 | 132 | 10.6 |
|  |  | Downstream | 30.0 | 23.0 | 2.57 | 92.8 | 30.6 |

*Supplementary Table S3. N2-N and O2 flux measurements and percent organic matter from sediment cores at each site. See excel file.*

| *Supplementary Table S4. Summary of linear regressions comparing sediment oxygen demand versus denitrification at upstream and downstream sites in the TFZ system on wet and dry core collection dates, as well as early and late summer experiments conducted in the TFZ in previous years. Asterisks denote statistical significance (‘ * ’ for p values < 0.10; ‘ ** ’ for p values < 0.05). There were no significant relationships from EST sediments and were therefore not reported.* | | | | | | |
| --- | --- | --- | --- | --- | --- | --- |
| Sampling period | Location | | Slope | $R^{2}$ | P value | Data source |
| Wet  *6/7/2021* | TFZ | Upstream | -0.0699 | 0.1402 | 0.7557 |  |
|  | TFZ | Downstream | 0.1807 | 0.9832 | 0.0828 * |  |
| Dry  *8/17/2021* | TFZ | Upstream | 0.1468 | 0.9996 | 0.0134 ** |  |
|  | TFZ | Downstream | 0.0206 | 0.5143 | 0.4909 |  |
| Early summer^a^ | TFZ | Upstream | -0.7729 | 0.4730 | 0.5172 | Von Korff et al. (2014) |
| Late summer^a^ | TFZ | Upstream | 0.3060 | 1.0000 | 0.0018 ** |  |
| Early summer^b^ | TFZ | Downstream | 0.1068 | 0.1876 | 0.3909 | Ensign et al. (2008) |
| Late summer^b^ | TFZ | Downstream | -0.0474 | 0.4944 | 0.1191 |  |
| ^a^ Early summer and late summer data are from experiments completed on 6/15/2021 and 9/2/2010, respectively. These data only include measurements from floodplain or riparian forest habitat.  ^b^ Early summer data were obtained by combining data collected on 5/16/2007 and 6/26/2007 Late summer data were obtained by combining data collected on 9/13/2006 and 8/7/2007. | | | | | | |
